# The National Dementia Workforce Study: Perspective From the National Institute on Aging

**DOI:** 10.1111/jgs.70065

**Published:** 2025-08-28

**Authors:** Priscilla Novak

**Affiliations:** ^1^ National Institute on Aging National Institutes of Health Bethesda Maryland USA

**Keywords:** Alzheimer's disease and related dementias (AD/ADRD), dementia care, dementia care quality, direct care workers, healthcare disparities, workforce retention

## Abstract

As the population of individuals living with Alzheimer's disease and related dementias (AD/ADRD) continues to grow, so does the need for a well‐prepared and stable dementia care workforce. The National Dementia Workforce Study (NDWS) represents a significant investment to understand the demographics, experiences, and magnitude and scope of challenges faced by the professional and direct care workforce providing care to people with dementia. This article discusses the NDWS's goals, data collection efforts, and data release. NDWS is the first large‐scale national survey of the dementia care workforce, encompassing physicians, advanced practice providers, nurses, direct care workers, and other health professionals across four key care settings: community‐based medical practices, nursing homes, assisted living communities, and home care. The study's innovative design allows for data linkage with Medicare claims and other health databases, providing a data infrastructure for understanding workforce factors that influence care quality and outcomes. The first datasets were released in July of 2025, and the data can be accessed at no cost. The linked NDWS‐Medicare datasets are available through a secure web portal for analysis. Findings from this analysis can answer novel questions that may shape the future of the dementia care workforce in the United States, ensuring that the growing population of people with dementia receives high‐quality care. As the study progresses, longitudinal results and annual data updates will continue to provide valuable insights, helping researchers, practitioners, and policymakers understand the workforce caring for people affected by dementia.

**Summary**

- Key points
  ○ The National Dementia Workforce Study (NDWS) datasets can be used by researchers to create a detailed analysis of the demographics and experiences of community clinicians and the direct care workforce, providing insights into the working conditions of doctors, physician assistants, and nurse practitioners, as well as the organizational conditions experienced by nursing and personal care staff working in nursing homes, assisted living facilities, and home care.
  ○ The datasets enable quantitative insights on the challenges faced by direct care workers, including low wages, high turnover rates, high risk of workplace injuries, and worker burnout and grief.
  ○ The NDWS's longitudinal design will enable researchers to track workforce changes over time, with the potential to render valuable insights into factors that contribute to workforce retention and turnover.
  ○ Insights from NDWS findings may elucidate relationships between worker conditions and patient safety and quality.
- Why does this paper matter?
  ○ The National Dementia Workforce Study is the first nationally representative survey available for analysis of the workforce and organizational factors influencing the quality of care delivered to people living with Alzheimer's disease and AD‐related dementias. The NDWS has significant potential to inform both practice and policy.
  ○ By identifying key organizational factors that influence the quality of dementia care, the study data can guide the development of targeted interventions to improve workforce training, support, and retention. Through innovative linkages, researchers will be able to explore the impact of paid leave, worker wages, and worker self‐efficacy on patient quality of care. The data can be used by researchers to create innovative secondary data analyses that may inform policies aimed at reducing workforce disparities, enhancing job satisfaction, and ultimately improving the care provided to people with dementia.

## 1 Introduction

The quality of the care received by people living with dementia matters, and the lives and experiences of paid caregivers are equally important [1]. Health care quality is widely defined as services which are safe, effective, patient‐centered, accessible, and timely, and efficient [2]. Since the 2011 release of the National Plan to Address Alzheimer's Disease [3], the National Institute on Aging has made significant strides toward advancing understanding of the quality of care delivered to people living with Alzheimer's disease (AD) and AD‐related dementias (ADRD), along with furthering the science of caregiving for people and families living with dementia. Through stakeholder engagement at the 2017 National Research Summit on Dementia Care and Services, the 2020 National Research Summit on Care, Services, and Supports for Persons with Dementia and Their Caregivers, and the 2023 National Research Summit on Care, Services, and Supports for Persons Living with Dementia and Their Care Partners/Caregivers, NIA has created a slate of research milestones intended to signal high priority areas for future study. These research milestones for AD/ADRD include the objective of improving our understanding of access to care and quality of services for people living with dementia, as well as elucidating key data about the dementia care workforce [3].

The National Dementia Workforce Study (NDWS) is responsive to ADRD research milestone 13.Y, which aims to assess the impact of dementia care workforce variation and composition on the health and well‐being outcomes of persons living with dementia, their family care partners, and the dementia care workers themselves. The Alzheimer's disease and related dementias milestones led to the creation of NDWS, which released its first year of data in 2025. Publicly available data files can be accessed by visiting NDWS.org.

NDWS data will enable examination of how differences in staffing patterns, shift work, employment status, and certification levels influence care quality and patient outcomes. The study aligns with the research milestone's objective of understanding care mix in systems. Additionally, the study data addresses the milestone's call to develop and test approaches that support all members of the dementia workforce across various professional levels and care settings. This includes addressing critical issues such as skills training, precarious employment, and promoting the replication and dissemination of successful workforce strategies. Data from the study will allow researchers to analyze how care organizational factors, team composition, and skill mix influence the effectiveness and sustainability of the dementia care workforce.

Prior studies documented that the workers caring for people living with dementia face issues like low pay, schedule variability, high‐turnover [4], and high rates of work‐related injuries. There is a shortage of both direct care workers as well as specialized geriatric professionals such as geriatric social workers, nurses, and physicians [5]. In order to meet the need for better data and measurement of the myriad challenges faced by dementia care workers, the National Institute on Aging awarded NDWS through a cooperative agreement in September of 2023. Now, with the release of the first wave of its survey data, researchers have access to datasets that can be used to fill gaps in knowledge on the supply side of the dementia care labor market and elucidate characteristics of optimal working conditions to improve the experience of work for the direct care workforce.

With the release of the initial NDWS datasets, researchers will now be able to better quantify the demographics and experiences of physicians, nurses, and direct care workers who provide daily care to individuals and families living with AD/ADRD. The NDWS datasets were proposed to cover four care settings: community‐based medical care, nursing homes, assisted living, and home care. These datasets aim to deepen our understanding of how institutions and professional caregivers support individuals with AD/ADRD and enable research on the care workforce, including the availability of skilled labor. The fair data principles of findability, accessibility, interoperability, and reusability inform the data sharing process that is operationalized for the datasets. Access to linked data can be requested through the National Institute on Aging's data LINKAGE program; see https://www.ndws.org/surveys‐and‐data/how‐to‐access‐data for detailed instructions.

## 2 Study Overview

The NDWS is the first large‐scale national survey of the dementia care workforce, encompassing physicians, advanced practice providers, nurses, direct care workers, and other health professionals across key care settings: office‐based healthcare, nursing homes, assisted living communities, and home care. The study's innovative design allows for data linkage with Medicare claims and other health databases, providing a data resource for understanding workforce factors that influence care quality and outcomes.

### 2.1 Community Providers

While studies from the Association of American Medical Colleges have long projected a shortfall in the primary care physician workforce, this data set will enable quantitative modeling on the academic preparation of community clinicians who care for people living with dementia. The survey of community clinicians captures data from physicians, physician assistants, and advanced practice nurses who provide care to people living with dementia [6, 7]. Thus, the survey data can be used for innovative studies on the characteristics of the workforce, as well as the use of specific treatments or types of care and how these vary by clinician and practice characteristics. The data can also address questions about community clinicians' confidence in diagnosing mild cognitive impairment and their attitudes about providing treatment for dementias in clinical practice. Linking the community clinician dataset to Medicare claims data in the secure environment of the NIA LINKAGE platform will allow for an examination of the concordance between clinician‐reported and claims‐based estimates of the proportion of patient panels with AD/ADRD.

The Clinician Survey seeks to capture the perspectives of doctors and advanced practice providers who provide care to community‐dwelling people with dementia. This is a critical perspective, given the shortage of geriatricians, neurologists, and geriatric psychiatrists in the United States [8, 9, 10]. While many surveys focus on physician [11], physician assistant [12], and nurse practitioner [13, 14, 15] demographics, knowledge, skills, and abilities, this survey is the first of its kind to capture the experience of licensed professionals with regard to their perceived capability to care for people living with dementia. This is especially important given recent developments in disease‐modifying drugs that target the early stages of the disease [16, 17], requiring prompt identification of cognitive decline [18] and accurate diagnosis of dementia type to effectively guide patients and their families to treatment and trials. The dataset on community clinicians allows researchers to examine geographic differences in the knowledge and experiences of community providers. By using a sampling strategy that includes physicians, nurse practitioners, and physician assistants across urban/suburban and rural settings, the dataset will enable researchers to develop a deeper understanding of whether differences in knowledge and practice exist across geographic regions of the US and among rural doctors and nurses. Prior research has suggested that rural populations are diagnosed later in their disease trajectory [19], live fewer days at home [19], and have less access to the advanced imaging currently required to monitor ADRD disease‐modifying treatments [20].

### 2.2 Direct Care Workforce

The Nursing Home Staff, Assisted Living Staff, and Home Care Staff surveys oversample individuals from populations that are underrepresented in AD/ADRD research who work in caregiving roles, including registered nurses, licensed practical nurses, nursing assistants, and home health aides [21]. These surveys will enable researchers to investigate how variations in care provided to individuals with AD/ADRD relate to the characteristics of care organizations and the direct care workforce, including those administering medications, assisting with feeding and toileting, or engaging patients in activities to alleviate symptoms of boredom or agitation.

As far as we know, this is the first survey of its kind to explore the demographics and experiences of caregivers who support families in remembering if medications have been taken, people and pets have been fed, and living quarters cleaned and picked up to preserve hygiene and safety. While these quotidian tasks are often taken for granted, they are the backbone of functioning that is required for safe and healthy lives and therefore essential to the well‐being of people living with dementia.

The surveys also capture knowledge and experience that can be used to study carers' abilities across different settings. Respondents from all three settings (nursing homes, assisted living, home care/health) answer questions about their understanding of dementia, perceived competence in communicating with and responding to the needs of dementia patients, and their interactions with families of those living with dementia. These surveys address direct care workers' awareness of behavioral management techniques, which prior studies have highlighted as a critical area for training [22, 23]. They also report on caregivers knowledge of care techniques in the end‐of‐life and maintaining safety for themselves and their residents, patients, and clients. Additional data include data elements on tenure with their current employer, the number of concurrent jobs they hold, their average weekly working hours, and their total number of years working in elder care.

Before the release of this survey data, there have been few nationally representative studies of the direct care workforce. While it was known that these workers often face financial difficulties and hold multiple jobs, it was unclear whether these issues stemmed from state‐level contexts or were reflective of broader national trends. With the availability of the detailed NDWS data, researchers can now provide more accurate insights into the financial challenges reported by direct care workers. These issues are crucial, as highlighted during the pandemic, where infection control and the availability of care workers were impacted. The survey data can inform workforce planning and the development of compensation strategies for employers and policymakers.

Understanding worker perspectives and job retention requires examining compensation structures and barriers to emotional and relational satisfaction. The surveys explore whether direct care workers have experienced physical or sexual assault [24], attempted assault [25], or discrimination based on race [26], ethnicity, religion, or national origin [27]. They also include questions about citizenship status and living situations involving immigration concerns [28], with options for workers to skip these questions if desired. Data on work‐related injuries and burnout are also captured, which are significant, given the high rates of back injuries and grief experienced by the workforce in recent years.

Retention in the direct care workforce has been a longstanding challenge [29, 30, 31], with high turnover rates [32]. Due to the low barriers to entry for these roles, employers such as fast‐food restaurants, daycares, and retail stores often compete for the same workers, offering—what for some workers—could be considered as more attractive pay, scheduling, and working conditions. The longitudinal aspect of the datasets will allow researchers using future data releases to estimate workforce exit rates and identify shared characteristics among workers with longer tenures. Facility and agency‐level data enable the study of organizational factors that may contribute to better retention. The datasets include elements on pay, scheduling, benefits, and tuition support. Importantly, the data from these surveys will be available at no cost to the research community, supporting studies that can inform policy, with robust privacy protections in place to ensure participants can respond without fear of reprisal from their current employer.

## 3 Looking Forward

Over the next 3 years, the National Dementia Workforce Study will continue to evolve, expanding its datasets with longitudinal data and enhancing the survey questionnaires to capture even more detailed insights about the dementia care workforce. This ongoing data collection will allow researchers to track changes in the workforce, such as job retention rates, shifts in workforce demographics, and evolving challenges faced by caregivers in various settings. Researchers wishing to use study data will be able to link survey data with Medicare claims and other health data, providing a richer and more comprehensive resource for understanding how workforce factors influence the quality of care and outcomes for people living with dementia.

Researchers who are interested in using the datasets are encouraged to learn more by visiting NDWS.org. The datasets gathered through the study have the potential to significantly improve the quality of care for Americans living with dementia, but only to the extent that they are used and the findings are disseminated to inform decision making. A robust data user community is vital to ensure that the data are analyzed and fulfill their highest purpose.

## 4 Summary

The NDWS is the first large‐scale national survey of its kind, allowing researchers to explore how workforce factors influence dementia care quality and outcomes. The data include information on the workforce's knowledge, skills, and experiences, as well as their challenges, such as job retention, financial struggles, and exposure to discrimination or workplace injuries. By linking survey data with Medicare claims and other health data, the study will allow researchers to develop a more nuanced understanding of the care provided to individuals with dementia and to inform policy and workforce planning. The NDWS will continue to evolve over the next 3 years, enhancing its datasets and potentially improving the quality and accessibility of dementia care in the United States.

## Author Contributions

The author meets the criteria for authorship. The author contributed to the conception, drafting, and final approval of the manuscript.

## Conflicts of Interest

The author is an employee of the National Institute on Aging.

## Linked Articles

This publication is part of the special collection of National Dementia Workforce Study. To view all articles under this special collection visit https://agsjournals.onlinelibrary.wiley.com/doi/toc/10.1111/(ISSN)1532‐5415.national‐workforce‐study.
